# 

**Published:** 1894-07-21

**Authors:** 


### CONTENTS.

**The Doctor Afloat** - 329

**Around the Hospitals** - 330

**On Modern Progress in Ophthalmic Medicine and Surgery.** By ROBERT BRUDENELL CARTER, F.R.C.S. - 331

**The Educational Treatment of Idiots, Imbeciles, and Feeble-Minded Children.** By G. E. SHUTTLEWORTH, B.A., M.D.—III. - 332

**Famous Poisoners in Fiction**—X. - 333

**The Field of Science** - 334

**Annotations.**—Who Supervises the M.O.H.?—Heat Stroke in England—What Epsom Does for Doctors - 335

**Notes and News** - 336

**Editor's Letter-Box** - 346

**Medical Progress and Hospital Clinics.**—

Diseases of the Ear - 337

MEDICAL PROGRESS.—Diseases of Children - 338

PROGRESS IN SURGERY.—The Operative Treatment of Cancer with Special Reference to Cancer of the Breast - 340

General Surgery - 341

**The Institutional Workshop:**

HOSPITAL CONSTRUCTION.—The New Derbyshire Royal Infirmary 343

THE STORY OF THE INSANE FROM YEAR TO YEAR- - 345

ASYLUMS IN INDIA.—VI. Burma: The Rangoon Asylum - 346

**Scraps and Gleanings** - 346

**Continental Notes from a Holiday Correspondent** - 347

**The Student of Medicine** - 347

### EXTRA SUPPLEMENT.—THE NURSING MIRROR.

**News from the Nursing World.**—Where Should the Flowers Go?—Workhouse Inspectors—Massage—Convalescent Home at Oxted—District Nursing at Camberwell—Nurses at Northampton—Increased Salaries—Woman Guardians for Newton Abbot—Workhouse Attendants—A Trained Nurse for Corwen—The Budapest Conference—Costly Beef Tea—A Memorial Hospital—Short Items clxi
**On General Nursing.**—XXI. Peritonitis (*continued*) clxiii
**Ambulance Work in Paris—Enthusiasm at Dartford** clxiii
**The Mechanism of Movement** IX.—The Foot clxiv
**The Universal Cookery and Food Association** clxiv
**The Matrons' Council—A Sad Accident** clxv
**Nursing in Chili—Novelties for Nurses** clxv
**Under Royal Patronage** clxv
**Notes from Victoria—Nursing in U.S.A.** clxvi
**For Reading to the Sick** clxvi
**The Workhouse Infirmary at Bishop Auckland** clxvii
**Nurses' Holidays.**—Southend—Where to Go clxviii
**The Muses' Looking Glass** clxix
**Everybody's Opinion—Appointments** clxx

